# The impact of continuous positive airway pressure combined with lifestyle intervention on patients with obstructive sleep apnea: a multilevel meta-analysis

**DOI:** 10.3389/fmed.2026.1748601

**Published:** 2026-02-20

**Authors:** Lele Yang, Zhikai Qin, Jiajun Lan, Tao Liu, Yang Zhu, Fuya Yao, Qilong Wang, Zheng Yi

**Affiliations:** 1 Capital University of Physical Education and Sports, Beijing, China; 2 School of Physical Education and Sport Science, Fujian Normal University, Fuzhou, China

**Keywords:** apnea-hypopnea index, continuous positive airway pressure, lifestyle intervention, multilevel meta-analysis, obstructive sleep apnea

## Abstract

**Background:**

Obstructive Sleep Apnea (OSA) is commonly managed with Continuous Positive Airway Pressure (CPAP), yet low adherence and insufficient metabolic improvements limit its effectiveness.

**Objective:**

This study aimed to systematically assess the effect of CPAP combined with lifestyle interventions on OSA severity, as measured by the Apnea-Hypopnea Index (AHI), and to explore potential moderators.

**Methods:**

This systematic review and meta-analysis followed the PRISMA 2020 guidelines. We identified randomized controlled trials (RCTs) in PubMed, Web of Science, the Cochrane Library, and Embase. Effect sizes were reported as mean differences (MD). Data were synthesized using multilevel random-effects models. Statistical heterogeneity was assessed with the I^2^ statistic and Cochran’s Q test. To identify potential moderators, we conducted sensitivity analyses, publication bias assessment, subgroup analyses, and meta-regression. The reliability of the evidence for each outcome was rigorously assessed using the GRADE framework.

**Results:**

Fourteen RCTs involving 1,623 patients were included. CPAP combined with lifestyle interventions significantly reduced AHI (MD = −9.99, 95% CI: −14.55 to −5.44, *p* < 0.001, GRADE: Moderate). Subgroup analyses showed greater benefits with multi-component lifestyle interventions (integrating diet, exercise, and behavioral strategies) (MD = −11.99, *p* < 0.001, GRADE: Moderate), intervention duration < 12 weeks (MD = −19.29, *p* < 0.001, GRADE: Low), moderate-to-severe OSA (MD = −11.55, *p* < 0.001, GRADE: Moderate), and BMI reduction of ≥ 5 kg/m^2^ (MD = −23.39, *p* < 0.001, GRADE: Low). Meta-regression analyses showed that most prespecified moderators were not statistically significant, whereas increasing age was associated with greater reductions in AHI (*β* = −1.12, *p* = 0.024).

**Conclusion:**

Moderate-quality evidence indicates that CPAP combined with lifestyle interventions improves AHI in patients with OSA, particularly those with moderate-to-severe OSA, obesity, or receiving multi-component lifestyle interventions. The evidence is limited by high heterogeneity and risk of bias.

**Systematic review registration:**

Systematic review registration: PROSPERO (CRD420251053389).

## Introduction

OSA has emerged as a significant global public health concern. Epidemiological data from 2019 showed that nearly one billion people aged 30–69 worldwide were affected, with China having the highest prevalence at 24.2% (1). The all-cause mortality risk among OSA patients was 4.2 times higher than that of the general population (2). This disease not only severely endangers individual health but also imposes a heavy economic burden—annual medical costs per patient in Europe ranged from €1,669 to €5,186 (3). Its dual impact on individuals and healthcare systems demands urgent attention.

The core characteristic of OSA is recurrent upper airway collapse, leading to intermittent hypoxemia, apnea/hypopnea events, and disruption of sleep architecture (4). However, this description simplified OSA to a purely biomechanical disorder, which is inconsistent with current insights. Recent studies have indicated that OSA etiology **is** strongly influenced by genetic factors and neurotransmitter regulation: Genetic variations in dopamine-related and serotonin pathway genes have been linked to OSA susceptibility and severity (5, 6); Dopamine and other abnormal neurotransmitters, along with TPH1 and other synthetic enzyme levels, are involved in OSA pathophysiology by regulating respiratory drive and upper airway muscle tone (7). These changes led to complications such as hypertension and metabolic dysfunction. Notably, 50–60% of obese individuals had comorbid OSA (8). OSA is also associated with sleep architecture abnormalities, dysregulation of biochemical marker (vitamin D, uric acid, magnesium) (9), circadian rhythm disruption, hypoxia-induced glucose metabolism disorders (10), and sex-related anatomical variations (e.g., hyoid bone position) that may guide personalized care (11). AHI (abnormal breathing events per hour) was the core diagnostic and severity indicator: AHI ≥ 5 confirmed diagnosis, with severity categorized as mild (5–14 events/h), moderate (15–30 events/h), and severe (>30 events/h) (12).

CPAP was the gold standard for OSA treatment, as it effectively improved airway patency, reduced AHI, and could decrease all-cause mortality by 37% (4). However, in clinical practice, low adherence and potential weight gain during treatment (with an average increase of 0.134 kg/m^2^ in BMI and 0.417 kg in body weight) (13) severely limited its therapeutic effect. For this reason, the American Academy of Sleep Medicine recommended in 2019 that lifestyle education and behavioral interventions be introduced simultaneously at the initial stage of CPAP treatment, especially for overweight/obese patients, to improve adherence and optimize outcomes (14).

Lifestyle interventions (including dietary adjustments, physical activity, and behavioral strategies) have demonstrated significant potential in OSA management by improving energy intake and lifestyle habits (15–17). Among these, multi-component interventions (such as diet + exercise, diet + exercise + behavioral sleep medicine) were more effective, with core elements including calorie restriction, aerobic/resistance training, and reduced sedentary behavior (18). Existing studies have confirmed that CPAP combined with weight loss could lower blood pressure (19) and that CPAP combined with exercise could enhance daytime alertness (20). However, most existing systematic reviews and meta-analyses focused on standalone lifestyle interventions and did not thoroughly examine the combined effects of CPAP and lifestyle interventions (21–24). Notably, significant heterogeneity existed in intervention composition, intensity, and control types across studies; the unique value of combined interventions relative to single regimens remained unclear, and the mechanisms by which lifestyle interventions improved CPAP adherence and synergistically alleviated OSA severity also required further clarification. Therefore, this study aimed to systematically evaluate the impact of CPAP combined with lifestyle interventions on AHI in patients with OSA, while exploring potential moderating factors, to provide evidence-based support for optimizing OSA treatment strategies.

## Methods

### Study design

This study was conducted as a systematic review and meta-analysis of RCTs, following the PRISMA (Preferred Reporting Items for Systematic Reviews and Meta-Analyses) guidelines (25). The study protocol was registered in PROSPERO on 23 March 2025 (registration number: CRD420251053389) and was updated on 23 January 2026 to ensure methodological transparency.

### Eligibility criteria

The inclusion and exclusion criteria were strictly defined using the PICO framework to ensure clarity and rigor. Eligible studies met the following criteria: Population: Adults aged ≥ 18 years with confirmed OSA (AHI ≥ 5), regardless of severity or comorbidities. Intervention: Experimental group receiving CPAP combined with lifestyle interventions (multi-component, e.g., diet + exercise; single-component, e.g., diet alone). Comparison: Control group receiving non-CPAP treatments, usual care, or CPAP alone; subgroup stratification and sensitivity analysis by comparator type were conducted to address heterogeneity. Outcome: Primary outcome was absolute AHI change (events/h) for clinical interpretability, with MD also reported for cross-study comparison. Studies were excluded if they involved surgical, pharmacological, or unspecified primary interventions; non-RCT designs (e.g., case reports, preprints); uncontrolled confounding co-interventions; or lacked extractable AHI data.

### Search strategy

A comprehensive literature search was conducted across multiple databases, including PubMed, Web of Science, Cochrane Library, and Embase. The search protocol was registered on 6 March 2025. To identify relevant studies, both Medical Subject Headings (MeSH) terms and free-text terms were used to capture key concepts related to the research topic. The search strategy included the following terms: (“obstructive sleep apnea” OR “sleep apnea, obstructive” OR “apneas obstructive”) AND (“continuous positive airway pressure” OR “CPAP”) AND (“lifestyle intervention” OR “lifestyle” OR “diet” OR “exercise” OR “physical activity”) AND (“apnea hypopnea index” OR “AHI”) AND (“Controlled Trial” OR “Randomised Controlled Trial” OR “RCT” OR “Clinical Trial”). See the Supplementary file. Additionally, the reference lists of eligible articles were manually reviewed to identify further studies.

### Study selection and data extraction

All records were imported into EndNote for deduplication. Two independent reviewers screened titles and abstracts, followed by full-text reviews to determine eligibility. Discrepancies were resolved by consulting a third reviewer. Data were independently collected using a standardized form, and any disagreements were resolved through discussion to ensure accuracy and reliability. When data were missing or presented only graphically, authors were contacted to request the necessary information. If this contact attempt was unsuccessful and the data were available only in graphical form, relevant data were extracted using WebPlotDigitizer 4.1 (https://automeris.io/WebPlotDigitizer) (26). This software has been shown to have high reliability and validity (27). If we failed to obtain the missing information, the specific study was excluded from the analysis.

### Statistical analysis

All meta-analyses were conducted in R using the meta, metafor, dmetar, and ggplot2 packages (28). Because AHI was consistently reported in identical units (events per hour) across the included studies, MD was used as the effect size to retain the original clinical units (29). The MD was calculated as the difference in mean AHI between the intervention and control groups. The magnitude of the MD was interpreted using the absolute reduction in AHI, in conjunction with established OSA severity classifications and their clinical implications for disease severity and symptom burden (12, 30).

The primary analyses were conducted using multilevel random-effects models via the rma.mv function, which accounted for correlations among multiple effect sizes from the same study (levels: sampling variance, within-study variance, and between-study variance) (31). Restricted maximum-likelihood (REML) estimation was used, and when heterogeneity was minimal (I^2^ < 50%), results from fixed-effects models were also included. Statistical heterogeneity was evaluated using Cochran’s Q test (*p* < 0.10 considered significant) and the I^2^ statistic, with values exceeding 50% indicating considerable heterogeneity (32). Publication bias was assessed using a funnel plot and Egger’s linear regression, and data with an asymmetric distribution were adjusted using the trim-and-fill technique (33). Outlier studies with strong influence on the model were identified using standardized residuals (|Z| > 2.5) or Cook’s distance (threshold > 3 times the mean) (34). To further verify the robustness of the results, the following sensitivity analyses were conducted: (1) leave-one-out analysis (Metainf function); (2) subgroup stratification tests to assess the sources of heterogeneity using categorical variables; (3) meta-regression analysis using REML to quantify the association between potential moderators and effect size (32).

### Risk of Bias (quality) assessment

The risk of bias in the included studies was assessed using the Cochrane Risk of Bias tool (RoB 2.0) for RCTs (35). This assessment emphasized several key factors: randomization, allocation concealment, blinding, incomplete data, and selective reporting. Two reviewers conducted the assessments independently, with a third reviewer available to resolve disagreements. Overall confidence in the evidence for primary outcomes (AHI) and all subgroup analyses was assessed using the GRADE framework, with specific criteria for downgrading. Findings were summarized in tables created with the GRADEpro GDT online tool (36).

## Result

### Study selection

**Figure 1:**
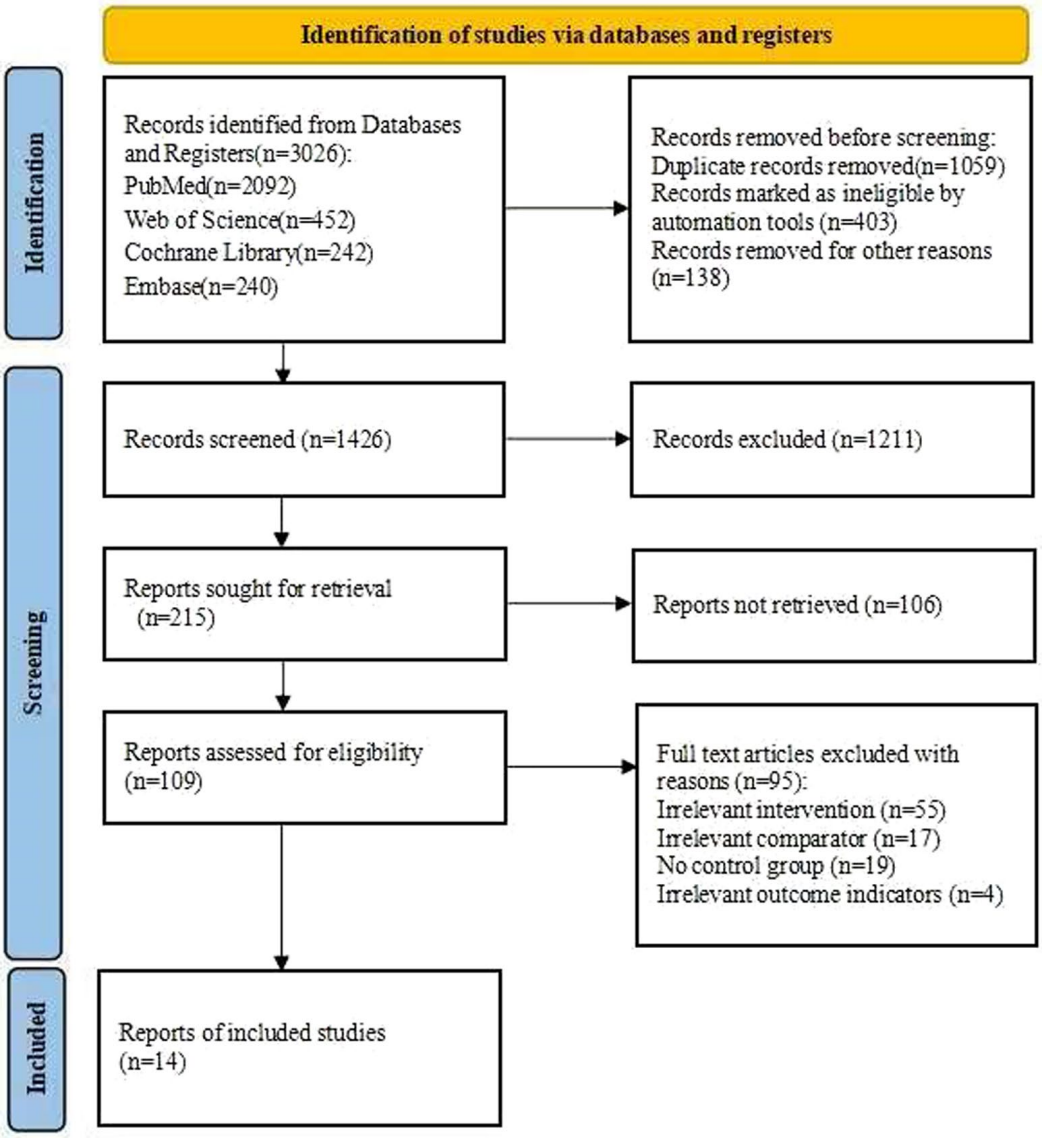
Flow diagram of the selection process.

A total of 3,181 studies were initially identified from four databases (Figure 1). Before formal screening, duplicate records were removed, some records were automatically marked as ineligible, and additional records were excluded for other reasons. Subsequently, 1,426 titles and abstracts were screened (at this stage, Cohen’s *κ* = 0.998), and 1,211 records were excluded. A total of 215 reports were sought for retrieval; of these, 106 could not be retrieved, and the remaining 109 underwent full-text eligibility assessment (at this stage, Cohen’s κ = 0.997) (see upplementary fil). Of these, 95 full-text articles were excluded for an irrelevant intervention, an irrelevant comparator, a lack of a control group, or irrelevant outcome indicators. Ultimately, 14 studies were included in this meta-analysis.

### Risk of Bias of included studies

**Figure 2:**
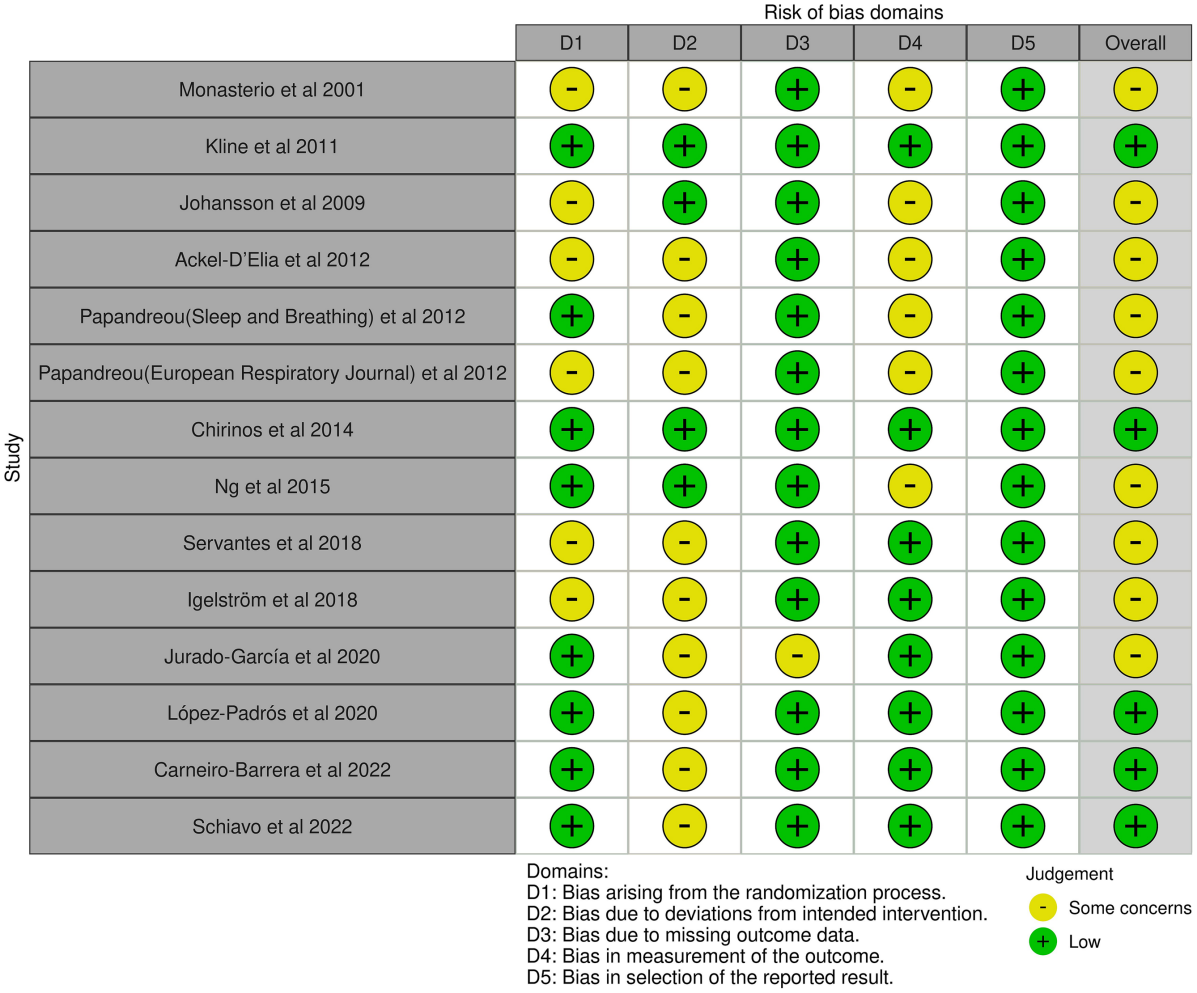
Risk of bias graph for included studies.

**Figure 3:**
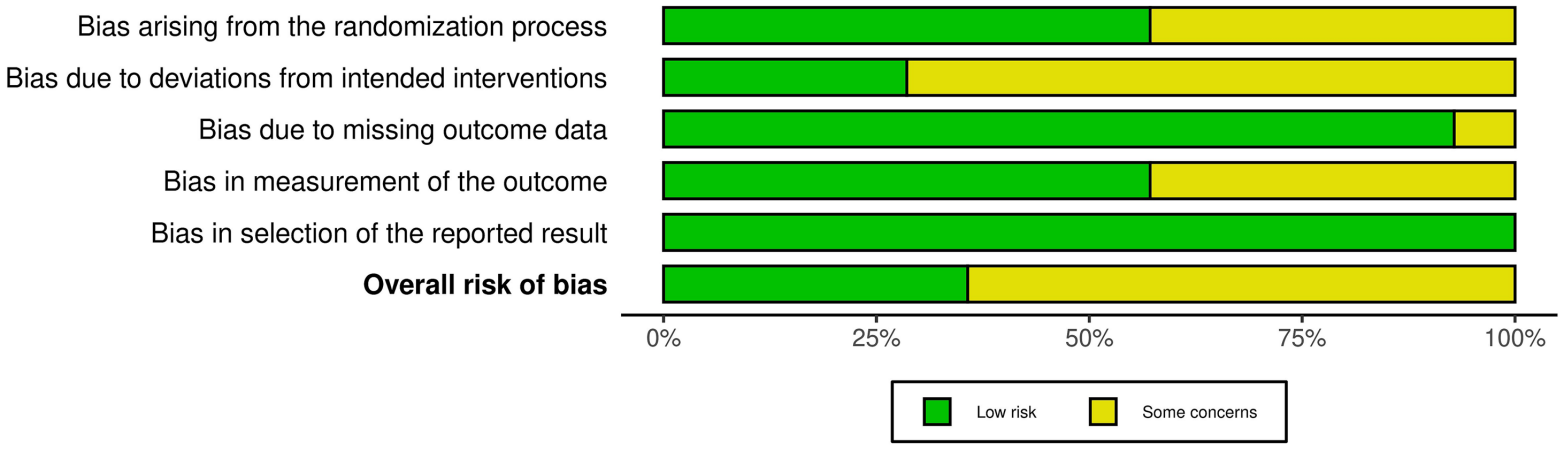
Risk of bias summary.

The risk of bias for the 14 included RCTs is shown in Figures 2, 3 (yellow “–” = Some concerns; green “+” = Low risk). Two reviewers independently assessed 5 RoB 2.0 domains (D1–D5); discrepancies were resolved through discussion or a third reviewer. Domain distribution: D1: some concerns for a subset; D2: some concerns; D3: 1 study with some concerns; D4: balanced low/some concerns; D5: all low risk. Overall, most studies had some concerns; only 5 (19, 37–40) had low risk.

Inter-rater agreement for each domain was as follows: For D1, the simple agreement rate was 71.4% (10/14), with Cohen’s *κ* = 0.43 and weighted κ = 0.60 (interpreted as “Moderate”); for D2, the simple agreement rate was 57.1% (8/14), with *κ* = 0.14 and weighted κ = 0.31 (interpreted as “Fair”); for D3, the simple agreement rate was 92.0% (13/14), with κ = 0.86 and weighted κ = 0.93 (interpreted as “Very Good”); for D4, the simple agreement rate was 71.4% (10/14), with Cohen’s κ = 0.43 and weighted κ = 0.60 (interpreted as “Moderate”); for D5, the simple agreement rate was 100% (14/14), with Cohen’s κ = 1.00 and weighted κ = 1.00 (interpreted as “Almost Perfect”) (see upplementary fil).

Overall, domains D3 and D5 had an extremely high proportion of “Low risk” ratings, indicating that the studies were relatively reliable regarding follow-up completeness and transparency in result reporting. In contrast, domains D1, D2, and D4 had more “Some concerns” ratings. Notably, D2 had the lowest inter-rater agreement (only “Fair”), reflecting relatively large discrepancies in judging this type of bias. These results indicate that some studies still had shortcomings in reporting randomization details, blinding participants and researchers, and ensuring intervention adherence. Based on this bias profile, we cautiously interpreted potential systematic bias related to D2 in the main and subgroup analyses and incorporated these biases into sensitivity analyses and GRADE evidence quality assessments.

### Study characteristics

Fourteen RCTs published between 2001 and 2022 were included (Table 1), covering countries such as Spain, Sweden, the United States, Greece, Brazil, China, and Italy. Participants were adults with OSA, aged 42 to 54 years, with baseline AHI of 20 to 45 events/h and baseline BMI of 29–36 kg/m^2^. Most patients had moderate-to-severe OSA, with obesity as the main health condition and a few were overweight. Intervention durations ranged from 4 to 48 weeks and included CPAP combined with dietary, exercise, or comprehensive lifestyle interventions. Control groups received CPAP alone, routine care, or CPAP plus standard lifestyle advice. Outcomes primarily focused on total AHI, with some studies also assessing supine, REM, and NREM AHI (19, 20, 37–48).

**Table 1:** Basic characteristics of included studies.

Author	Country	Age	Total sample	Intervention cycle (wk)	Baseline AHI (events/h)	Change in BMI (kg/m^2^)	OSA severity	Health condition	Outcome	Intervention mode
Monasterio et al. (41)	Spain	54 ± 9	125	26	20 ± 6	0–3	Mild	Obesity	Total AHI	CT + CPAP vs. CT
Johansson et al. (42)	Sweden	49 ± 7.3	63	9	37 ± 15	≥5	Moderate-to-severe	Obesity	Total AHI, Supine AHI	liquid very low energy diet+CPAP vs. usual diet+CPAP
Kline et al. (38)	America	46.9 ± 1.2	43	12	29.1 ± 7.8	N/A	Moderate-to-severe	Overweight	Total AHI, Supine AHI, NREM AHI, REM AHI	CPAP+Exercise training vs. Stretching control + CPAP
Papandreou et al. (43)	Greece	48.9 ± 12.7	40	26	>15	3–5	Moderate-to-severe	Obesity	Total AHI, REM AHI	Mediterranean Diet+Physical Activity+CPAP vs. Prudent Diet +Physical Activity+CPAP
Papandreou et al. (44)	Greece	48.1 ± 12.4	21	26	>15	3–5	Moderate-to-severe	Obesity	Total AHI	Mediterranean diet+CPAP vs. prudent diet+CPAP
Ackel-D’Elia et al. (20)	Brazil	48.95 ± 8.48	32	13	>15	N/A	Moderate-to-severe	Overweight	Total AHI	sleep hygiene+exercise+CPAP vs. sleep hygiene+CPAP
Chirinos et al. (19)	America	N/A	181	24	42.7 ± 23.2	N/A	Moderate-to-severe	Obesity	Total AHI	CPAP + Weight-loss intervention vs. CPAP
Ng et al. (45)	China	51.7 ± 9.2	104	48	43.0 ± 20.0	0–3	Moderate-to-severe	Obesity	Total AHI, Supine AHI	LM*P* + CPAP vs. CPAP
Servantes et al. (46)	Brazil	54.5 ± 8.5	33	12	28.46	N/A	Moderate-to-severe	Obesity	Total AHI	exercise+CPAP vs. CPAP
Igelström et al. (47)	Sweden	54.9 ± 11.8	86	26	43.5 ± 20.7	N/A	Moderate-to-severe	Overweight	Total AHI	CPAP+BSM intervention targeting physical activity+eating behavior vs. CPAP+advice about weight loss
López-Padrós et al. (39)	Brazil	49 ± 6.7	34	48	>30	0–3	Severe	Obesity	Total AHI, Supine AHI, NREM AHI, REM AHI	IWLP+CPAP vs. receiving standard lifestyle recommendations+CPAP
Jurado-García et al. (48)	Spain	51 ± 8.2	68	26	28 ± 16.2	0–3	Moderate-to-severe	Obesity	Total AHI	CPAP + Usual care + Graduated Walking Program vs. Usual care + CPAP
Carneiro-Barrera et al. (40)	Spain	49 ± 7.3	63	8	37 ± 15	0–3	Moderate-to-severe	Obesity	Total AHI, REM AHI, NREM AHI	interdisciplinary weight loss and lifestyle intervention combined with usual-care+CPAP vs. CPAP
Schiavo et al. (37)	Italy	42 ± 13.7	70	4	≥30	≥5	Severe	Obesity	Total AHI	Low-calorie ketogenic diets +CPAP vs. CPAP

CT, conservative treatment—sleep hygiene and weight loss; LMP, lifestyle modification program; IWLP, intensive weight-loss program; BSM, behavioral sleep medicine; AHI, apnea-hypopnea index; BMI, body mass index; OSA, obstructive sleep apnea; CPAP, continuous positive airway pressure; REM, rapid eye movement sleep; NREM, non-rapid eye movement sleep; OSA severity classification criteria: Mild (AHI = 5 ~ 14); Moderate (AHI = 15 ~ 30); Severe (AHI> 30); “N/A” indicates that specific data for the indicator was not clearly provided in the original article; “0–3,” “3–5,” and “≥5” are BMI change ranges corresponding to the grouping criteria in the subgroup analysis; In the intervention mode, the left side of “vs” represents the experimental group intervention plan, and the right side represents the control group intervention plan.

### Multilevel Meta-analysis results

**Figure 4:**
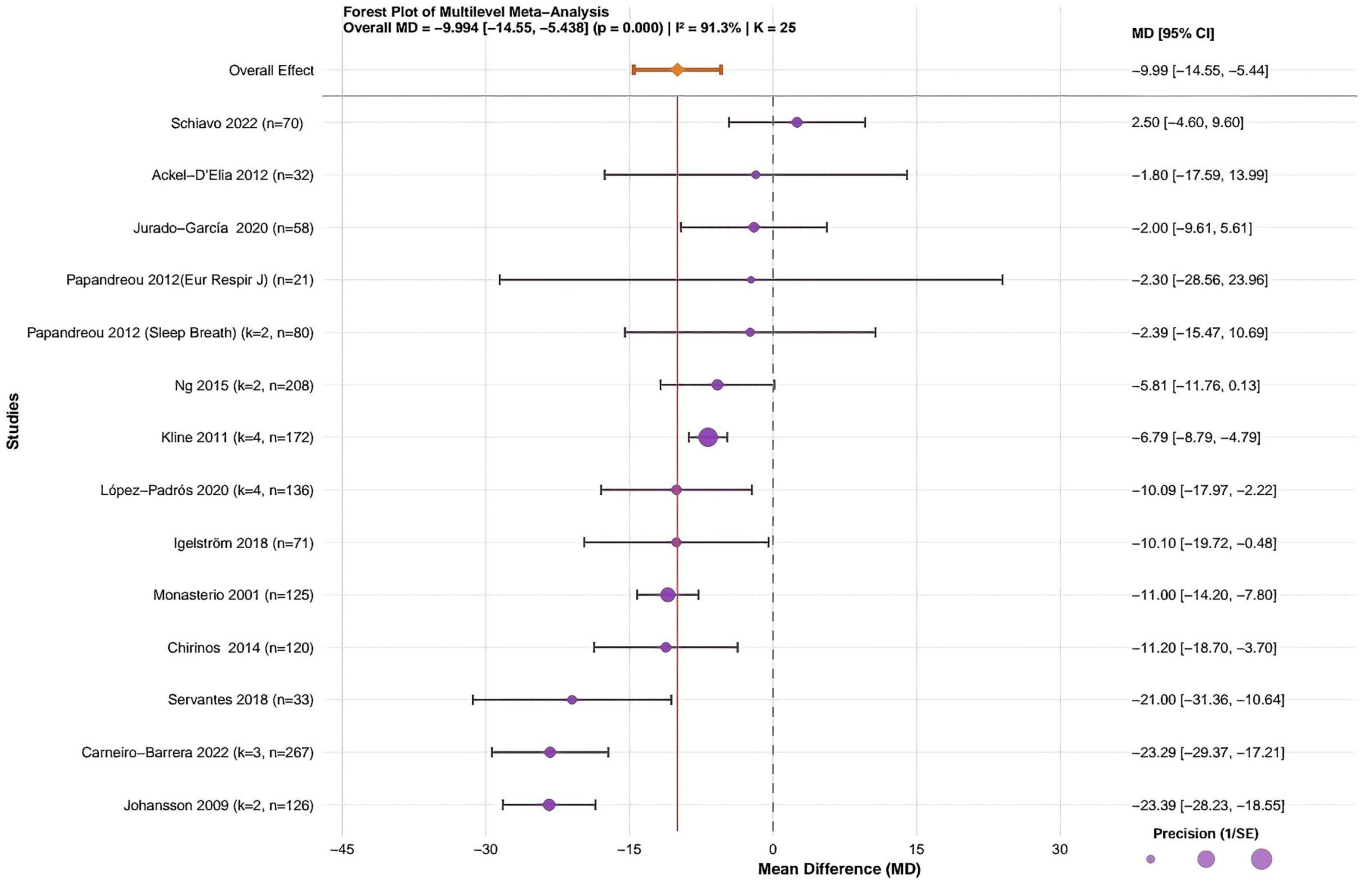
Forest plot of the multilevel meta-analysis for the effect of CPAP combined with lifestyle intervention on AHI in patients with OSA. Forest plot shows the mean difference (MD) and 95% confidence interval (CI) of AHI changes for individual studies and the overall combined effect. Horizontal lines represent individual studies (square size, study weight; line length = 95% CI). Vertical dashed line, no effect (MD = 0); 95% CI not crossing this line indicates statistical significance. *K*, number of effect sizes; *n*, total sample size; CPAP, continuous positive airway pressure; OSA, obstructive sleep apnea; AHI, apnea-hypopnea index.

This study systematically assessed the effects of CPAP combined with lifestyle interventions on patients with OSA. A total of 14 randomized controlled trials involving 1,623 participants were included to evaluate changes in AHI. Substantial heterogeneity was observed across studies (I^2^ = 91.3%, *p* < 0.001). The pooled analysis showed that CPAP combined with lifestyle interventions was associated with a statistically significant overall reduction in AHI (MD = −9.99, 95% CI: −14.55 to −5.44, *p* < 0.001) (Figure 4). Because I^2^ was greater than 50% (I^2^ = 91.3%, *p* < 0.001), Egger’s regression was used to evaluate potential publication bias. Egger’s regression is a statistical method used to detect publication bias in meta-analysis, primarily to assess whether study results were affected by small-sample effects (49). The multilevel Egger’s test indicated significant asymmetry (intercept t = −2.348, *p* = 0.019), suggesting the possible influence of small-study effects or selective reporting (see Supplementary file). Sensitivity analysis in meta-analysis is a statistical method used to assess the robustness and reliability of the results by determining the extent of the impact on overall outcomes when key parameters are altered or specific studies are excluded (50). Sensitivity analysis showed that the top 5 studies with the most significant impact on the pooled effect size did not cause significant deviation, confirming the overall pooled effect size’s stability (see Supplementary file).

Subsequently, standardized residuals (|Z| > 2.5) and Cook’s distance (threshold > 3 times the mean) were used to identify influential outliers in the model (51). The results indicated that the studies by Servantes et al. (46) and Jurado-García et al. (48). Studies had Cook’s distances exceeding the threshold but did not exceed the standardized residual threshold and were therefore not excluded (see Supplementary file). Furthermore, the trim-and-fill analysis showed that no studies needed to be imputed (labeled “No studies imputed”), and the adjusted effect size was identical to that of the original multilevel model (MD = −9.99). This finding further corroborates the robustness of the meta-analytic conclusions (see supplementary file). In addition, the small-sample-size test confirmed the statistical significance of the pooled effect size (Estimate = −9.99, SE = 2.33; t-statistic = −4.29, df = 12.1, *p* = 0.00103) (see Supplementary file). According to the GRADE assessment, moderate-quality evidence indicates a potential benefit of CPAP combined with lifestyle interventions in reducing AHI among patients with OSA. However, the strength of this evidence is constrained by risk of bias and pronounced heterogeneity (see Supplementary file).

Therefore, fourteen randomized controlled trials with 1,623 participants were synthesized using a random-effects model due to substantial heterogeneity across studies (I^2^ = 91.3%, *p* < 0.001). The pooled estimate showed an absolute reduction in AHI with the combined intervention (MD = −9.99, 95% CI: −14.55 to −5.44) (Figure 4).

### Subgroup analysis

This study explored potential moderators through subgroup analyses, including (1) magnitude of BMI reduction, (2) intervention type (experimental/control), (3) intervention duration, (4) OSA severity, and (5) type of AHI measurement (Table 2).

**Table 2:** Subgroup analysis of CPAP Combined with lifestyle intervention on obstructive sleep apnea patients.

Subgroup category	N (K)	MD (95% CI)	Heterogeneity, I^2^	Subgroup sample size	GRADE judgment	*P* value
Change in BMI						
0–3 kg/m^2^	4 (11)	−12.02 (−16.72, −7.32)	62.40%	726	Moderate	0.000***
3–5 kg/m^2^	3 (4)	0.43 (−7.19, 8.06)	0.00%	171	Very low	0.911
≥5 kg/m^2^	1 (2)	−23.39 (−28.23, –18.55)	0.00%	126	low	0.000***
Experimental group dominant intervention type						
CPAP + Exercise intervention	4(7)	−7.29 (−10.65, −3.94)	60.40%	305	Moderate	0.000***
CPAP + Dietary intervention	3 (4)	−13.33 (−27.80,1.14)	86.50%	217	Very low	0.071
CPAP + Comprehensive lifestyle intervention	7 (14)	−11.99(−15.60, −8.39)	42.80%	1,005	Moderate	0.000***
Control group dominant intervention type						
CPAP alone	6 (9)	−12.65(−19.07, −6.23)	73.60%	713	Moderate	0.000***
CPAP + Routine lifestyle intervention	4 (8)	−6.40 (−10.87, −1.93)	0.00%	370	Moderate	0.005**
Intervention cycle						
< 12 weeks	3 (6)	−19.29(−27.35, −11.23)	77.70%	385	Low	0.000***
≥ 12 weeks	11 (19)	−7.97 (−9.98, −5.95)	27.90%	1,142	Moderate	0.000***
OSA severity						
Moderate-to-Severe	11 (19)	−11.55 (−15.20, −7.90)	73.70%	1,222	Moderate	0.000***
Severe	2 (5)	−6.32 (−14.20, 1.55)	32.60%	206	Very Low	0.115
AHI type						
Total AHI	14 (14)	−9.39 (−13.84, −4.95)	78.20%	963	Moderate	0.000***
Supine AHI	4 (4)	−12.14 (−20.73, −3.55)	66.20%	244	Low	0.006**
NREM AHI	3 (3)	−11.60 (−22.81, −0.38)	78.90%	140	Low	0.043*
REM AHI	4 (4)	−14.46 (−21.91, −7.02)	40.90%	180	Moderate	0.000***

N(K), total number of studies (K) and total sample size (N) included in each subgroup; MD, mean difference; 95% CI, 95% confidence interval; Heterogeneity was evaluated by I^2^ statistic: I^2^ < 25%, low heterogeneity; 25–50%, moderate heterogeneity; > 50%, high heterogeneity; *p < 0.05; **p < 0.01; ***p < 0.001; CPAP, continuous positive airway pressure; OSA, obstructive sleep apnea; AHI, apnea-hypopnea index; REM, rapid eye movement; NREM, non-rapid eye movement; “Comprehensive lifestyle intervention” refers to combined dietary and exercise interventions; “Routine lifestyle intervention” denotes standard health advice without structured dietary or exercise guidance.

In the experimental group, CPAP combined with comprehensive lifestyle intervention (MD = −11.99, 95% CI: −15.60 to −8.39, *p* < 0.001, GRADE: Moderate) and CPAP combined with exercise intervention (MD = −7.29, 95% CI: −10.65 to −3.94, *p* < 0.001, GRADE: Moderate) significantly improved AHI; CPAP combined with dietary intervention did not reach statistical significance (*p* = 0.071, GRADE: Very Low). In the control group, both CPAP alone (MD = −12.65, 95% CI: −19.07 to −6.23, *p* < 0.001, GRADE: Moderate) and CPAP plus routine lifestyle intervention (MD = −6.40, 95% CI: −10.87 to −1.93, *p* = 0.005, GRADE: Moderate) achieved significant AHI improvement. Regarding intervention duration, both < 12 weeks (MD = −19.29, 95% CI: −27.35 to −11.23, *p* < 0.001, GRADE: Low) and ≥ 12 weeks (MD = −7.97, 95% CI: −9.98 to −5.95, *p* < 0.001, GRADE: Moderate) significantly reduced AHI, with a more pronounced effect in the < 12 weeks subgroup. For OSA severity, moderate-to-severe patients showed significant improvement (MD = −11.55, 95% CI: −15.20 to −7.90, *p* < 0.001, GRADE: Moderate), whereas severe patients did not (*p* = 0.115, GRADE: Very Low). Regarding BMI reduction, the 0–3 kg/m^2^ (MD = −12.02, 95% CI: −16.72 to −7.32, *p* < 0.001, GRADE: Moderate) and ≥ 5 kg/m^2^ (MD = −23.39, 95% CI: −28.23 to −18.55, *p* < 0.001, GRADE: Low) subgroups showed significant AHI improvement, with no significant effect in the 3–5 kg/m^2^ subgroup (*p* = 0.911, GRADE: Very Low). Among AHI measurement types, Total AHI (MD = −9.39, 95% CI: −13.84 to −4.95, *p* < 0.001, GRADE: Moderate), supine AHI (MD = −12.14, 95% CI: −20.73 to −3.55, *p* = 0.006, GRADE: Low), NREM AHI (MD = −11.60, 95% CI: −22.81 to −0.38, *p* = 0.043, GRADE: Low), and REM AHI (MD = −14.46, 95% CI: −21.91 to −7.02, *p* < 0.001, GRADE: Moderate) all showed significant reductions.

These results indicated that CPAP combined with exercise or comprehensive lifestyle intervention, shorter intervention durations (more effective in < 12 weeks), moderate-to-severe OSA, and BMI reductions of 0–3 kg/m^2^ or ≥ 5 kg/m^2^ were more conducive to optimizing AHI reduction in OSA patients; REM AHI was the most sensitive to intervention. The most effective subgroups had moderate GRADE evidence quality.

### Meta-regression analysis

**Figure 5:**
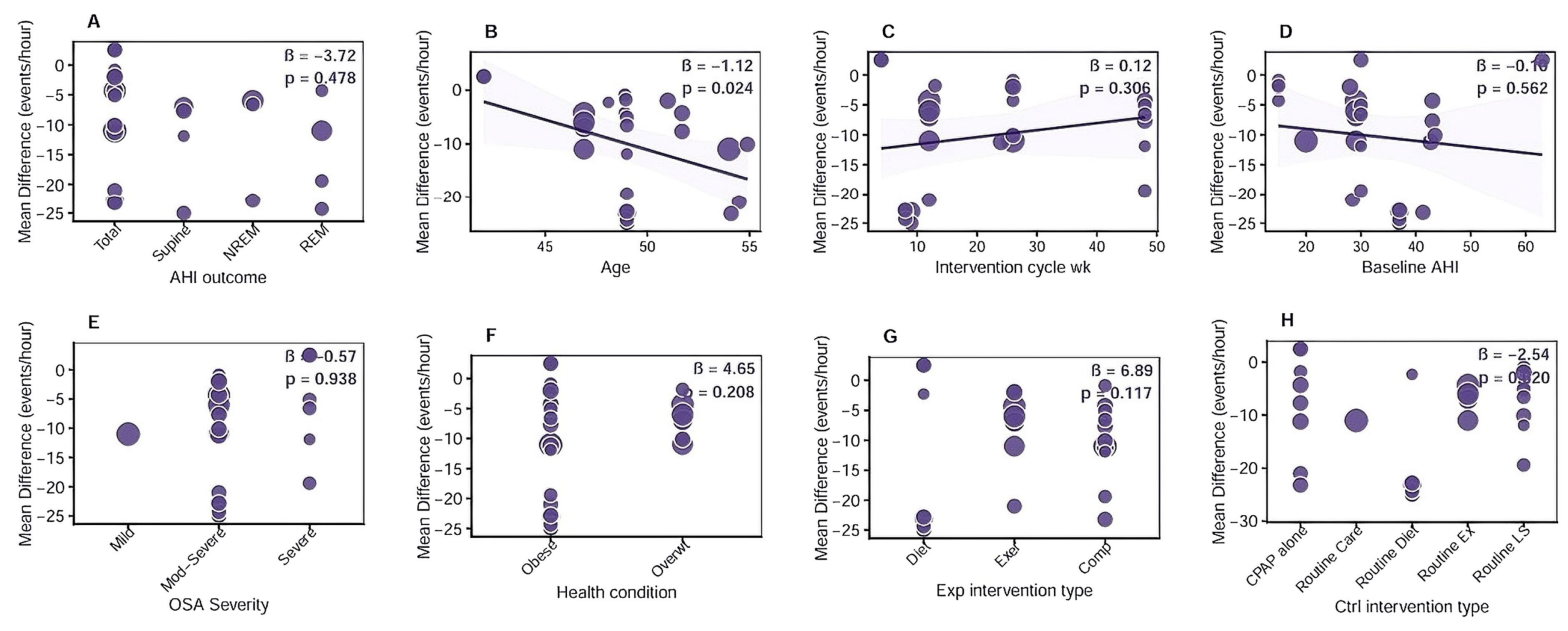
Meta-regression analysis of the effect of CPAP combined with lifestyle intervention on AHI in patients with OSA. **(A)** AHI outcome type: categorical variable including total AHI, REM AHI, NREM AHI, and supine AHI; **(B)** Age: continuous variable (mean/SD, years) of participants in included studies; **(C)** Intervention cycle: continuous variable (weeks) of the combined intervention duration; **(D)** Baseline AHI: continuous variable (events/h) of participants’ AHI before intervention; **(E)** OSA severity: categorical variable classified as mild (AHI = 5–14), moderate (AHI = 15–30), and severe (AHI > 30); **(F)** Health condition: ategorical variable including routine health status and comorbid-related health status; **(G)** Experimental intervention type: ategorical variable including CPAP + exercise, CPAP + dietary, and CPAP + comprehensive lifestyle intervention; **(H)** Control intervention type: ategorical variable including CPAP alone and CPAP + routine lifestyle intervention. *β*, regression coefficient; CPAP, continuous positive airway pressure; OSA, obstructive sleep apnea; AHI, apnea-hypopnea index; REM, rapid eye movement; NREM, non-rapid eye movement; MD, mean difference.

To explore potential sources of heterogeneity, random-effects meta-regression analyses using REML estimation were conducted to assess associations between eight pre-specified moderators and the pooled effect size (MD). The moderators included AHI outcome type, age, intervention cycle, baseline AHI, OSA severity, health condition, experimental intervention type, and control intervention type. As shown in Figure 5, most moderators did not significantly explain between-study heterogeneity (all *p* > 0.05), including AHI outcome type (*β* = −3.72, *p* = 0.478), intervention cycle (β = 0.12, *p* = 0.306), baseline AHI (β = −0.16, *p* = 0.562), OSA severity (β = −0.57, *p* = 0.938), health condition (β = 4.65, *p* = 0.208), experimental intervention type (β = 6.89, *p* = 0.117), and control intervention type (β = −2.54, *p* = 0.386).

Notably, age was significantly associated with the pooled effect size (β = −1.12, *p* = 0.024), indicating that greater reductions in AHI were observed with increasing age. This suggested that older patients tended to derive greater benefit from CPAP combined with lifestyle interventions. However, the explanatory power of the meta-regression analysis was limited, likely due to the relatively small number of included studies and substantial heterogeneity in intervention protocols.

## Discussion

This meta-analysis demonstrated that continuous positive airway pressure combined with lifestyle interventions reduced OSA severity, as reflected by a clinically meaningful reduction in the AHI (MD = −9.99, 95% CI: −14.55 to −5.44, *p* < 0.001). CPAP therapy provides rapid improvement in AHI by mechanically stabilizing the upper airway and reducing intermittent hypoxemia (4, 52–54), whereas lifestyle interventions primarily improve OSA severity by gradually modifying upstream pathophysiological factors, including excess adiposity and metabolic dysfunction, with AHI reduction closely linked to the degree of weight loss (55, 56). When combined, these interventions may act synergistically by providing immediate airway control and longer-term metabolic and inflammatory regulation, particularly in multi-component programs that integrate diet, exercise, and behavioral strategies (18, 19, 22).

Subgroup analysis stratified by disease severity showed that patients with moderate-to-severe OSA derived significant benefit from the combined intervention (MD = −11.55, *p* < 0.001), whereas no statistically significant improvement was observed among patients with severe OSA (*p* = 0.115). This finding may be attributable to a greater prevalence of fixed or irreversible upper airway anatomical abnormalities in severe OSA, which could limit responsiveness to lifestyle modification (57). Subgroup analyses stratified by BMI change showed that reductions of ≥5 kg/m^2^ were associated with the most significant improvements in AHI (*p* < 0.001), consistent with previous evidence that a weight loss of at least 10% could reduce AHI by approximately 40–50% (55, 56). Furthermore, subgroup analyses by AHI outcome type showed that REM sleep–related AHI was most responsive to the combined intervention (MD = −14.46, *p* < 0.001), which may reflect the pronounced reduction in upper airway muscle tone during REM sleep and the heightened sensitivity of this sleep stage to metabolic and neuromuscular improvements (58). In addition, subgroup analyses by control intervention type indicated that studies using CPAP alone as the control condition reported a larger reduction in AHI (MD = −12.65). This finding may reflect heterogeneity in control group design, as some control groups included participants with lower baseline AHI or better CPAP adherence than those in the combined intervention groups, underscoring the need for more standardized control protocols in future trials.

Although subgroup analyses suggested that REM sleep–related AHI showed the most considerable absolute reduction across AHI outcome types, meta-regression did not identify AHI subtype as a statistically significant moderator of the intervention effect. Overall, most examined moderators—including AHI outcome type and control intervention type—failed to account for the substantial between-study heterogeneity, indicating that the observed subgroup differences should be interpreted descriptively rather than as definitive effect modifiers. Notably, age was the only moderator significantly associated with AHI reduction in the meta-regression model, with greater treatment effects observed with increasing age. This finding may reflect age-related differences in OSA pathophysiology, whereby older patients tend to present with more collapsible upper airways, reduced neuromuscular compensation, and greater dependence on mechanical stabilization and metabolic improvement (59–61). Consequently, the combined effects of CPAP and lifestyle interventions—through enhanced airway patency, improved metabolic regulation, and reduced systemic inflammation—may produce more pronounced reductions in AHI among older individuals (62, 63). However, given the limited number of included studies and residual heterogeneity, this age-related association should be interpreted with caution.

## Conclusion

This multilevel meta-analysis of 14 randomized controlled trials involving 1,623 patients provides moderate-quality evidence that continuous positive airway pressure, combined with lifestyle interventions, significantly reduced obstructive sleep apnea severity. Compared with control conditions, the combined intervention produced a clinically meaningful reduction in AHI (MD = −9.99, 95% CI: −14.55 to −5.44; *p* < 0.001), despite substantial between-study heterogeneity (I^2^ = 91.3%). Subgroup analyses showed greater AHI reductions with multi-component lifestyle interventions (MD = −11.99), in patients with moderate-to-severe OSA (MD = −11.55), and in those achieving substantial BMI reduction (≥5 kg/m^2^; MD = −23.39). REM sleep–related AHI showed the most significant improvement (MD = −14.46), whereas no statistically significant benefit was observed in severe OSA. Meta-regression indicated that most prespecified moderators did not significantly explain heterogeneity; however, increasing age was associated with greater AHI reduction (*β* = −1.12, *p* = 0.024). Collectively, these findings support integrating structured lifestyle interventions with CPAP therapy in OSA management, while underscoring the need for standardized intervention protocols and longer-term randomized trials.

### Limitations and future directions

Several limitations should be acknowledged. First, the number of included studies was relatively small, and follow-up durations were generally short, which may have limited the evaluation of long-term treatment effects. Second, variability in lifestyle intervention components and intensity contributed to substantial heterogeneity across studies. Future large-scale, long-term randomized controlled trials using standardized lifestyle intervention protocols are warranted to confirm these findings and further explore individualized treatment strategies across different patient populations.

## Data Availability

The original contributions presented in the study are included in the article/Supplementary material, further inquiries can be directed to the corresponding author/s.
