# Supplementary material for: The impact of continuous positive airway pressure combined with lifestyle intervention on patients with obstructive sleep apnea: a multilevel meta-analysis
Source: Front Med (Lausanne). 2026 Feb 20;13:1748601. doi: 10.3389/fmed.2026.1748601 (PMC12963349; doi:10.3389/fmed.2026.1748601)
Supplement: Supplementary file 1 [file Supplementary_file_1.docx]

Contents

[Supplementary document Retrieval Strategy 1](#_Toc2970)

[Quality Assessment 3](#_Toc1699)

[Study Selection Consistency and Cohen’s κ 4](#_Toc19419)

[Inter-Rater Agreement Results for Each ROB2 Domain 4](#_Toc3878)

[Egger’s Test Funnel Plot for Publication Bias Assessment. 5](#_Toc10912)

[Influence diagnostics for included studies using standardized residuals and Cook’s distance. 6](#_Toc3475)

[Figure of the Initial Sensitivity Analysis. 6](#_Toc21577)

[Top 5 Studies with the Greatest Impact on the Pooled Effect Size in Leave-One-Out Sensitivity Analysis 7](#_Toc17001)

[CR2 Robust Variance Test Results (Satterthwaite Correction) 7](#_Toc15987)

[Trim-and-Fill Funnel Plot. 8](#_Toc3972)

[Summary of Findings — Effects of CPAP Combined with Lifestyle Intervention on AHI in Patients with Obstructive Sleep Apnea 8](#_Toc17272)

## Supplementary document Retrieval Strategy

| **Data** | **Query** | **Results** |
| --- | --- | --- |
| PubMed | ((("apnea hypopnea index"[Title/Abstract] OR "AHI"[All Fields]) AND ("CPAP"[Title/Abstract] OR "continuous positive airway pressure"[Title/Abstract]) AND ("sleep apnea, obstructive"[MeSH Terms] OR ("sleep apnea, obstructive"[MeSH Terms] OR ("sleep"[All Fields] AND "apnea"[All Fields] AND "obstructive"[All Fields]) OR "obstructive sleep apnea"[All Fields] OR ("apneas"[All Fields] AND "obstructive"[All Fields] AND "sleep"[All Fields]) OR "apneas obstructive sleep"[All Fields]) OR ("sleep apnea, obstructive"[MeSH Terms] OR ("sleep"[All Fields] AND "apnea"[All Fields] AND "obstructive"[All Fields]) OR "obstructive sleep apnea"[All Fields] OR ("obstructive"[All Fields] AND "sleep"[All Fields] AND "apneas"[All Fields]) OR "obstructive sleep apneas"[All Fields]) OR ("sleep apnea, obstructive"[MeSH Terms] OR ("sleep"[All Fields] AND "apnea"[All Fields] AND "obstructive"[All Fields]) OR "obstructive sleep apnea"[All Fields] OR ("sleep"[All Fields] AND "apneas"[All Fields] AND "obstructive"[All Fields]) OR "sleep apneas obstructive"[All Fields]) OR ("sleep apnea, obstructive"[MeSH Terms] OR ("sleep"[All Fields] AND "apnea"[All Fields] AND "obstructive"[All Fields]) OR "obstructive sleep apnea"[All Fields] OR ("apnea"[All Fields] AND "obstructive"[All Fields] AND "sleep"[All Fields]) OR "apnea obstructive sleep"[All Fields]) OR ("sleep apnea, obstructive"[MeSH Terms] OR ("sleep"[All Fields] AND "apnea"[All Fields] AND "obstructive"[All Fields]) OR "obstructive sleep apnea"[All Fields] OR ("sleep"[All Fields] AND "apnea"[All Fields] AND "hypopnea"[All Fields] AND "syndrome"[All Fields]) OR "sleep apnea hypopnea syndrome"[All Fields]) OR ("obstructive sleep apnoea syndrome"[All Fields] OR "sleep apnea, obstructive"[MeSH Terms] OR ("sleep"[All Fields] AND "apnea"[All Fields] AND "obstructive"[All Fields]) OR "obstructive sleep apnea"[All Fields] OR ("obstructive"[All Fields] AND "sleep"[All Fields] AND "apnea"[All Fields] AND "syndrome"[All Fields]) OR "obstructive sleep apnea syndrome"[All Fields]) OR ("obstructive sleep apnoea"[All Fields] OR "sleep apnea, obstructive"[MeSH Terms] OR ("sleep"[All Fields] AND "apnea"[All Fields] AND "obstructive"[All Fields]) OR "obstructive sleep apnea"[All Fields] OR ("obstructive"[All Fields] AND "sleep"[All Fields] AND "apnea"[All Fields])) OR ("sleep apnea, obstructive"[MeSH Terms] OR ("sleep"[All Fields] AND "apnea"[All Fields] AND "obstructive"[All Fields]) OR "obstructive sleep apnea"[All Fields] OR ("syndrome"[All Fields] AND "obstructive"[All Fields] AND "sleep"[All Fields] AND "apnea"[All Fields]) OR "syndrome obstructive sleep apnea"[All Fields]) OR ("sleep apnea, obstructive"[MeSH Terms] OR ("sleep"[All Fields] AND "apnea"[All Fields] AND "obstructive"[All Fields]) OR "obstructive sleep apnea"[All Fields] OR ("syndrome"[All Fields] AND "sleep"[All Fields] AND "apnea"[All Fields] AND "obstructive"[All Fields]) OR "syndrome sleep apnea obstructive"[All Fields]) OR ("sleep apnea, obstructive"[MeSH Terms] OR ("sleep"[All Fields] AND "apnea"[All Fields] AND "obstructive"[All Fields]) OR "obstructive sleep apnea"[All Fields] OR ("sleep"[All Fields] AND "apnea"[All Fields] AND "syndrome"[All Fields] AND "obstructive"[All Fields]) OR "sleep apnea syndrome obstructive"[All Fields]) OR ("sleep apnea, obstructive"[MeSH Terms] OR ("sleep"[All Fields] AND "apnea"[All Fields] AND "obstructive"[All Fields]) OR "obstructive sleep apnea"[All Fields] OR "osahs"[All Fields]) OR ("upper airway resistance sleep apnoea syndrome"[All Fields] OR "sleep apnea, obstructive"[MeSH Terms] OR ("sleep"[All Fields] AND "apnea"[All Fields] AND "obstructive"[All Fields]) OR "obstructive sleep apnea"[All Fields] OR ("upper"[All Fields] AND "Airway"[All Fields] AND "resistance"[All Fields] AND "sleep"[All Fields] AND "apnea"[All Fields] AND "syndrome"[All Fields]) OR "upper airway resistance sleep apnea syndrome"[All Fields]))) OR ("upper airway resistance sleep apnoea syndrome"[All Fields] OR "sleep apnea, obstructive"[MeSH Terms] OR ("sleep"[All Fields] AND "apnea"[All Fields] AND "obstructive"[All Fields]) OR "obstructive sleep apnea"[All Fields] OR ("upper"[All Fields] AND "Airway"[All Fields] AND "resistance"[All Fields] AND "sleep"[All Fields] AND "apnea"[All Fields] AND "syndrome"[All Fields]) OR "upper airway resistance sleep apnea syndrome"[All Fields])) AND ((("life style"[MeSH Terms] OR ("life"[All Fields] AND "style"[All Fields]) OR "life style"[All Fields] OR "lifestyle"[All Fields] OR "lifestyles"[All Fields]) AND ("intervention s"[All Fields] OR "interventions"[All Fields] OR "interventive"[All Fields] OR "methods"[MeSH Terms] OR "methods"[All Fields] OR "intervention"[All Fields] OR "interventional"[All Fields])) OR ("diet"[MeSH Terms] OR "diet"[All Fields]) OR ("exercise"[MeSH Terms] OR "exercise"[All Fields] OR ("physical"[All Fields] AND "activity"[All Fields]) OR "physical activity"[All Fields])) | 2092 |
| Web of science | (((TS=(sleep apnea, obstructive OR sleep apnea, obstructive OR sleep apnea obstructive OR obstructive sleep apnea" OR "apneas obstructive sleep OR apneas obstructive sleep OR sleep apnea, obstructive OR sleep apnea obstructive OR obstructive sleep apnea OR obstructive sleep apneas OR obstructive sleep apneas OR sleep apnea, obstructive OR sleep apnea obstructive OR obstructive sleep apnea OR sleep apneas obstructive OR sleep apneas obstructive OR sleep apnea, obstructive OR sleep apnea obstructive OR obstructive sleep apnea OR apnea obstructive sleep OR apnea obstructive sleep OR sleep apnea, obstructive OR sleep apnea obstructive OR obstructive sleep apnea OR sleep apnea hypopnea syndrome OR sleep apnea hypopnea syndrome OR obstructive sleep apnoea syndrome OR sleep apnea, obstructive OR sleep apnea obstructive OR obstructive sleep)) AND TS=(lifestyle intervention OR diet OR physical activity)) AND TS=(CPAP OR continuous positive airway pressure)) | 452 |
| Cochrane library | (CPAP OR continuous positive airway pressure)AND(lifestyle intervention OR diet OR physical activity)AND(sleep apnea, obstructive OR sleep apnea, obstructive OR sleep apnea obstructive OR obstructive sleep apnea OR apneas obstructive sleep OR apneas obstructive sleep OR sleep apnea, obstructive OR sleep apnea obstructive OR obstructive sleep apnea OR obstructive sleep apneas OR obstructive sleep apneas OR sleep apnea, obstructive OR sleep apnea obstructive OR obstructive sleep apnea OR sleep apneas obstructive OR sleep apneas obstructive OR sleep apnea, obstructive OR sleep apnea obstructive OR obstructive sleep apnea OR apnea obstructive sleep OR apnea obstructive sleep OR sleep apnea, obstructive OR sleep apnea obstructive OR obstructive sleep apnea OR sleep apnea hypopnea syndrome OR sleep apnea hypopnea syndrome OR obstructive sleep apnoea syndrome OR sleep apnea, obstructive OR sleep apnea obstructive OR obstructive sleep | 242 |
| Embase | (CPAP OR continuous positive airway pressure)AND(lifestyle intervention OR diet OR physical activity) AND (sleep apnea, obstructive OR sleep apnea, obstructive OR sleep apnea obstructive OR obstructive sleep apnea OR apneas obstructive sleep OR apneas obstructive sleep OR sleep apnea, obstructive OR sleep apnea obstructive OR obstructive sleep apnea OR obstructive sleep apneas OR obstructive sleep apneas OR sleep apnea, obstructive OR sleep apnea obstructive OR obstructive sleep apnea OR sleep apneas obstructive OR sleep apneas obstructive OR sleep apnea, obstructive OR sleep apnea obstructive OR obstructive sleep apnea OR apnea obstructive sleep OR apnea obstructive sleep OR sleep apnea, obstructive OR sleep apnea obstructive OR obstructive sleep apnea OR sleep apnea hypopnea syndrome OR sleep apnea hypopnea syndrome OR obstructive sleep apnoea syndrome OR sleep apnea, obstructive OR sleep apnea obstructive OR obstructive sleep apnea) | 240 |

## Quality Assessment

| **Study** | **D1** | **D2** | **D3** | **D4** | **D5** | **Overall** |
| --- | --- | --- | --- | --- | --- | --- |
| Monasterio et al 2001 | Some concerns | Some concerns | Low | Some concerns | Low | Some concerns |
| Kline et al 2011 | Low | Low | Low | Low | Low | Low |
| Johansson et al 2009 | Some concerns | Low | Low | Some concerns | Low | Some concerns |
| Ackel-D’Elia et al 2012 | Some concerns | Some concerns | Low | Some concerns | Low | Some concerns |
| Papandreou(Sleep and Breathing) et al 2012 | Low | Some concerns | Low | Some concerns | Low | Some concerns |
| Papandreou(European Respiratory Journal) et al 2012 | Some concerns | Some concerns | Low | Some concerns | Low | Some concerns |
| Chirinos et al 2014 | Low | Low | Low | Low | Low | Low |
| Ng et al 2015 | Low | Low | Low | Some concerns | Low | Some concerns |
| Servantes et al 2018 | Some concerns | Some concerns | Low | Low | Low | Some concerns |
| Igelström et al 2018 | Some concerns | Some concerns | Low | Low | Low | Some concerns |
| Jurado-García et al 2020 | Low | Some concerns | Some concerns | Low | Low | Some concerns |
| López-Padrós et al 2020 | Low | Some concerns | Low | Low | Low | Low |
| Carneiro-Barrera et al 2022 | Low | Some concerns | Low | Low | Low | Low |
| Schiavo et al 2022 | Low | Some concerns | Low | Low | Low | Low |

## Study Selection Consistency and Cohen’s κ

1. Title/Abstract Screening (Records Screened = 1426)

- Independent 2×2 table (T.L. vs Y.Z.): both include = 215; T.L. include / Y.Z. exclude = 0; T.L. exclude / Y.Z. include = 0; both exclude = 1211 (total n = 1426; a + b + c = 215, consistent with the PRISMA flowchart).
- Observed agreement: Po​ = (215+1211)/1426≈0.9993
- Expected agreement: Pe ​≈ 0.8236
- Cohen’s κ ≈ 0.998 (more precisely 0.9980)
- Approximate standard error: SE ≈ 0.003
- 95% CI: κ ≈ 0.992–1.004 (upper bound truncated at 1.00 as customary when the normal approximation slightly exceeds 1)
- Z ≈ 332.7 (p≪0.001)
- Interpretation (Landis & Koch): κ≈0.998, indicating almost perfect agreement.

2. Full-Text Screening (Reports Assessed = 109)

Independent 2×2 table (T.L. vs Y.Z.): both include = 14; T.L. include / Y.Z. exclude = 0; T.L. exclude / Y.Z. include = 0; both exclude = 95 (total n = 109; a + b + c = 14, consistent with the PRISMA flowchart).

- Observed agreement: P_o_ ​= (14+95)/109 ≈ 0.9991
- Expected agreement: P_e_​ ≈ 0.8872
- Cohen’s κ ≈ 0.997 (more precisely 0.9970)
- Approximate standard error: SE ≈ 0.005
- 95% CI: κ ≈ 0.987–1.007 (upper bound truncated at 1.00 as customary when the normal approximation slightly exceeds 1)
- Z ≈ 199.4 (p ≪ 0.001)

Interpretation (Landis & Koch): κ ≈ 0.997, indicating almost perfect agreement.

## Inter-Rater Agreement Results for Each ROB2 Domain

The table below displays the simple agreement rate, Cohen's Kappa, and Weighted Kappa calculated separately for each domain (D1-D5).

| **Domain** | **Simple Agreement Rate** | **Cohen’s κ** | **Weighted κ** | **Strength of Agreement** |
| --- | --- | --- | --- | --- |
| D1 (Randomization process) | 71.4% (10/14) | 0.43 | 0.60 | Moderate |
| D2 (Deviations from intended interventions) | 57.1% (8/14) | 0.14 | 0.31 | Fair |
| D3 (Missing outcome data) | 92.0% (13/14) | 0.86 | 0.93 | Very Good |
| D4 (Measurement of the outcome) | 71.4% (10/14) | 0.43 | 0.60 | Moderate |
| D5 (Selection of the reported result) | 100% (14/14) | 1.00 | 1.00 | Almost Perfect |


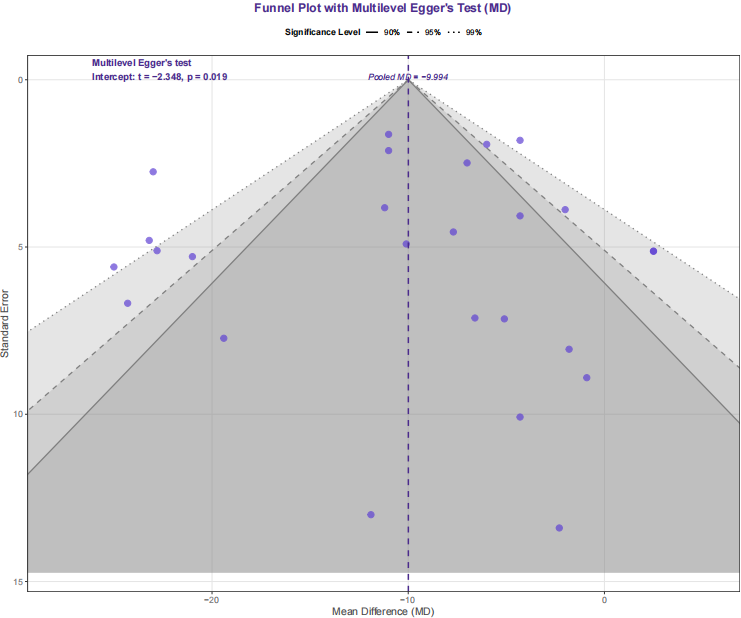


## Egger’s Test Funnel Plot for Publication Bias Assessment.


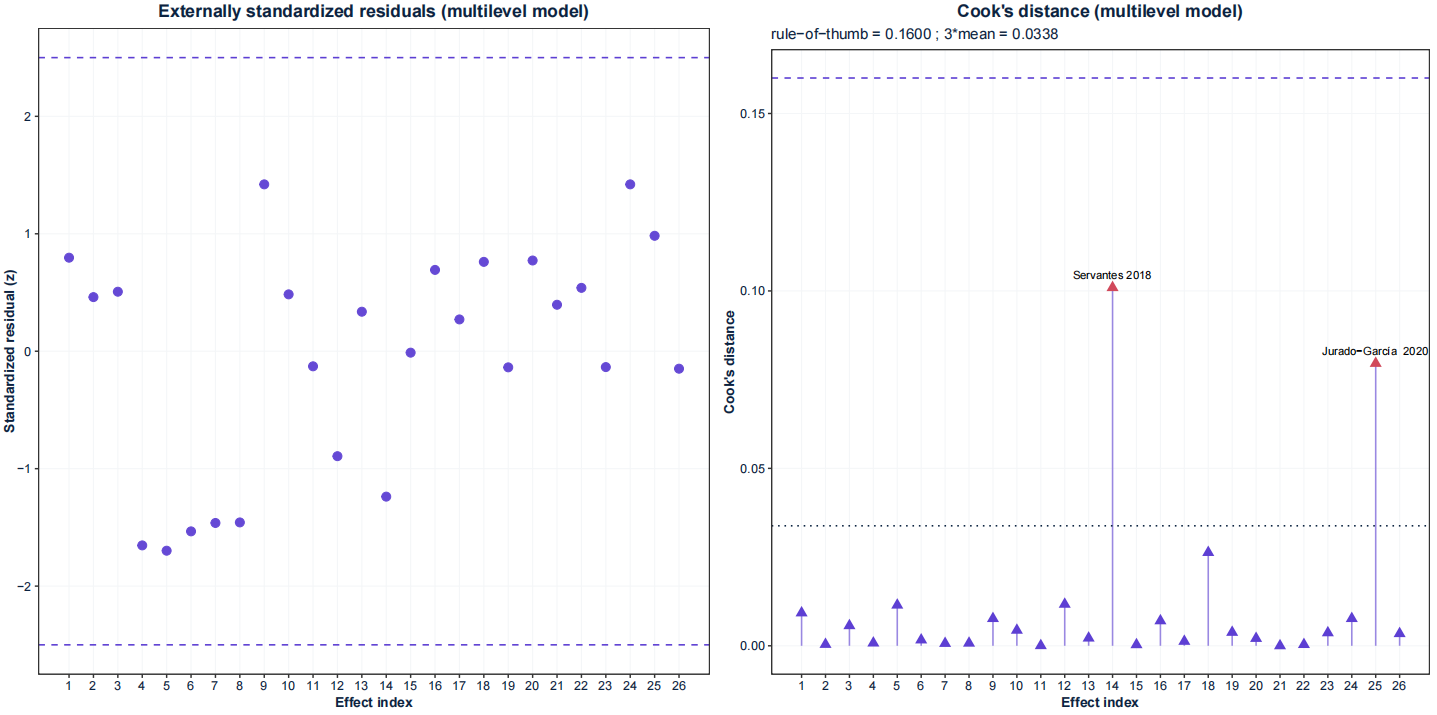


## Influence diagnostics for included studies using standardized residuals and Cook’s distance.


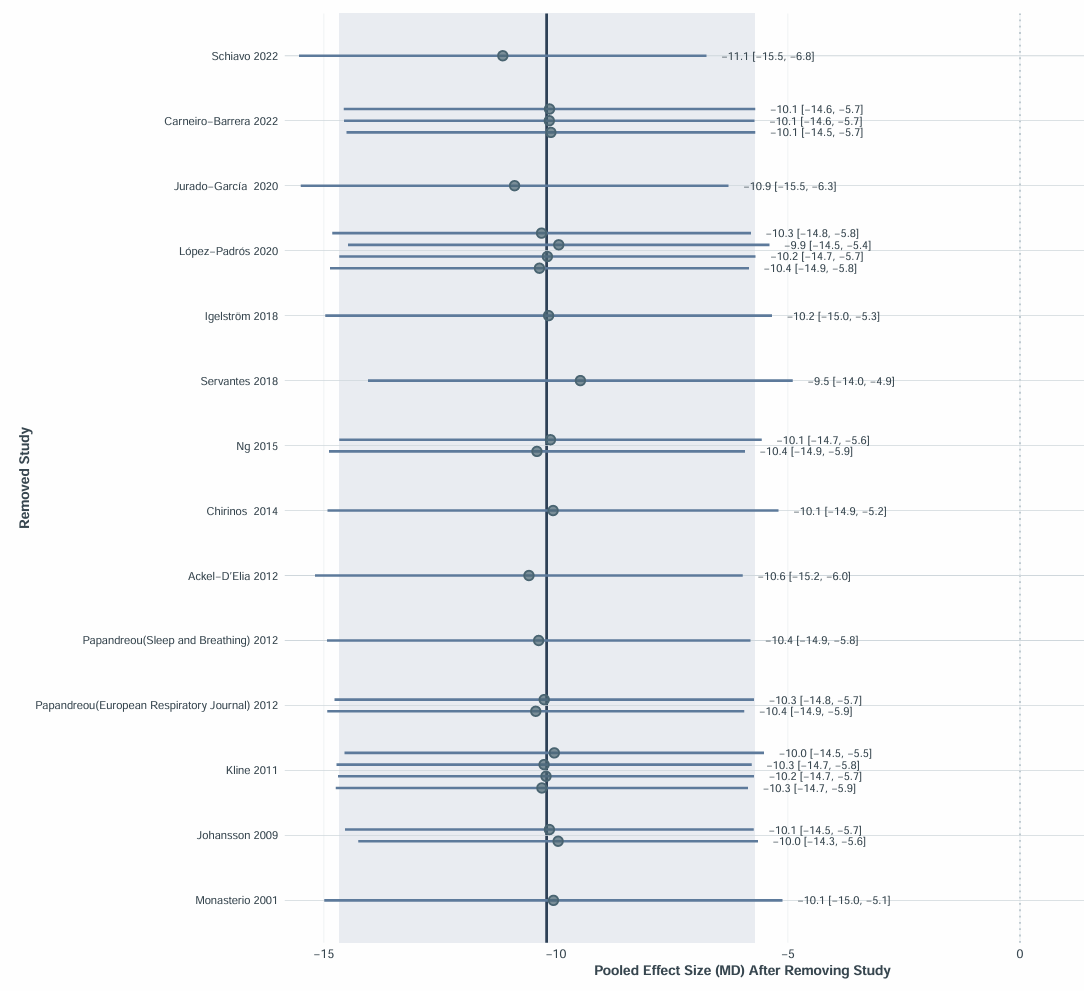


## Figure of the Initial Sensitivity Analysis.

## Top 5 Studies with the Greatest Impact on the Pooled Effect Size in Leave-One-Out Sensitivity Analysis

| **Study** | **Pooled Effect Size After Removal (95%CI)** | **Deviation from Overall Effect Size** | **Significantly Deviated from Overall Effect Size** |
| --- | --- | --- | --- |
| Servantes 2018 | -9.255 (-13.907, -4.604) | +0.738 | No |
| Jurado-García 2020 | -10.650 (-15.374, -5.925) | -0.656 | No |
| Ackel-D’Elia 2012 | -10.371 (-15.073, -5.668) | -0.377 | No |
| López-Padrós 2020 | -9.742 (-14.353, -5.132) | +0.251 | No |
| Johansson 2009 | -9.745 (-14.161, -5.329) | +0.249 | No |

Notes: The overall pooled effect size was MD = -9.990 (95%CI: -14.550, -5.440), P < 0.001; Deviation is calculated as "effect size after removal - overall effect size": positive values indicate an increased effect size after removal, while negative values indicate a decreased effect size; None of the high-impact studies showed significant deviation from the overall effect size (0/26 studies with significant deviation), indicating good stability of the pooled effect size in this study.

## CR2 Robust Variance Test Results (Satterthwaite Correction)

| **Coefficient** | **Estimate** | **SE (Standard Error)** | **Null Value** | **t-statistic** | **Satterthwaite df** | **Satterthwaite-corrected p-value** | **Test Direction** | **Statistical Significance** |
| --- | --- | --- | --- | --- | --- | --- | --- | --- |
| Intercept | -9.99 | 2.33 | 0 | -4.29 | 12.1 | 0.00103 | Two-sided | Significant |

Notes: This test was based on CR2 robust variance estimation, with degrees of freedom corrected by the Satterthwaite method for robust analysis suitable for small samples; Criterion for statistical significance: p < 0.05. The present result (p = 0.00103) confirms the stable statistical significance of the pooled effect size.


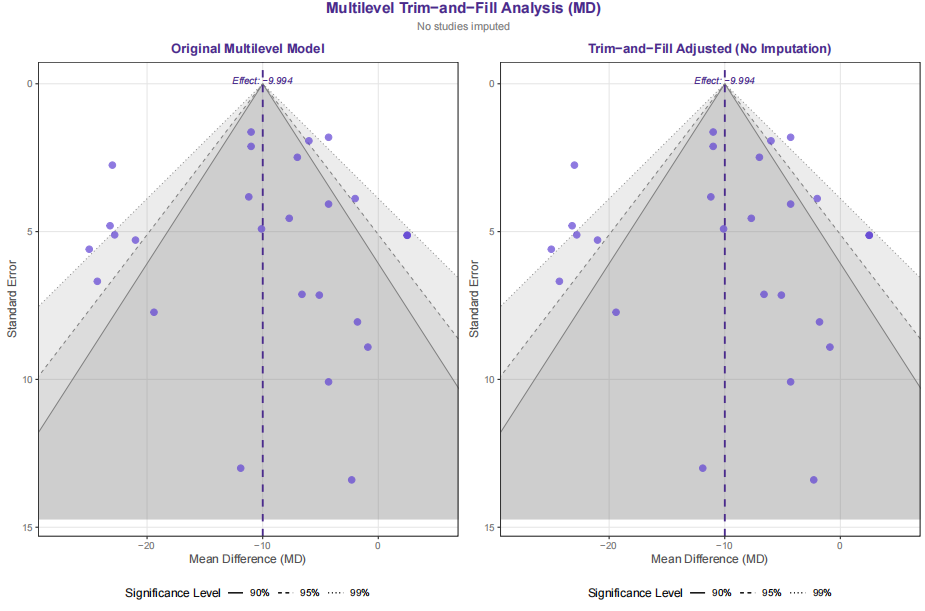


## Trim-and-Fill Funnel Plot.

## Summary of Findings — Effects of CPAP Combined with Lifestyle Intervention on AHI in Patients with Obstructive Sleep Apnea

| GRADE Domain | Judgment | Rationale (Strictly Based on the Original Article) |
| --- | --- | --- |
| Risk of Bias | Moderate (Downgraded by 1 level) | Among the 14 included RCTs, domains D1 (randomization process, simple agreement rate = 71.4%, Cohen’s κ = 0.43) and D4 (measurement of the outcome, simple agreement rate = 71.4%, Cohen’s κ = 0.43) were rated as "Moderate" agreement, and D2 (deviations from intended interventions, simple agreement rate = 57.1%, Cohen’s κ = 0.14) as "Fair" agreement. D3 (missing outcome data, 92.0% low risk) and D5 (selection of the reported result, 100% low risk) had high reliability; no study was rated as high risk. |
| Inconsistency | Serious (Downgraded by 1 level) | Heterogeneity was extremely high (I² = 91.3%, P < 0.001). Subgroup analysis explored factors such as BMI reduction, intervention type, and duration, identifying subgroups with more obvious effects, but meta-regression failed to confirm significant moderators, and heterogeneity was not fully explained. |
| Indirectness | Not downgraded | The population (adults with OSA, AHI ≥ 5 events/hour), intervention (CPAP combined with lifestyle interventions), comparator (non-CPAP treatments, usual care, or CPAP alone), and outcome (absolute change in AHI) were directly aligned with the research question, with no concerns regarding applicability. |
| Imprecision | Not downgraded | A total of 14 studies (1623 participants) were included, with a pooled effect size of MD = -9.99 (95%CI: -14.55 to -5.44, P < 0.001). The confidence interval did not cross 0, and sensitivity analysis (leave-one-out, trim-and-fill) showed stable results, confirming robustness. |
| Publication Bias | Not downgraded | Egger’s test indicated significant publication bias (Intercept: t = -2.348, p = 0.019), but trim-and-fill analysis showed no need for imputing missing studies, and the adjusted effect size was consistent with the original (MD = -9.99), suggesting no substantial impact on the overall result. |
